# Supplementary material for: Protective Efficacy of Subunit Vaccine Expressing Rv0976c Against Tuberculosis
Source: Vaccines (Basel). 2025 Aug 17;13(8):872. doi: 10.3390/vaccines13080872 (PMC12390289; doi:10.3390/vaccines13080872)
Supplement: Supplementary file 1 [file vaccines-13-00872-s001.zip › Supplementary figure legend.pdf]

Figure S1. Serum antibody levels of Rv0976c. Rv0976c (A), Ag85A (B) and ESAT-6 (C) antigens were coated onto Elisa plates, respectively, and the serum collected from TB patients and healthy controls were diluted at 1:100 as primary antibodies to detect the respect antibody levels in human serum. Data were plotted as mean ± SEM (n=30, 20). *, $p<0.05$, unpaired student's t-test.

Figure S2 Detection of pcDNA-Ag85a, pcDNA-FadD21, pcDNA-PPE18, pcDNA-PPE19, pcDNA-PPE50, pVax-*Rv0976c* and pVax-Ag85a expression in 293T cells. Control represents the 293T cell lysate without transfection of any plasmids. The expression of each construct in mammalian cells was confirmed by transfecting 293T or HeLa cells (ATCC) using the lipofectamine LTX kit (Life technologies, Lot No: 13778-075), followed by Western blot analysis using antibody against His tag. The expression status of each plasmid is shown in the Figure S2.
