# Supplementary material for: Protective Efficacy of Subunit Vaccine Expressing Rv0976c Against Tuberculosis
Source: Vaccines (Basel). 2025 Aug 17;13(8):872. doi: 10.3390/vaccines13080872 (PMC12390289; doi:10.3390/vaccines13080872)
Supplement: Supplementary file 1 [file vaccines-13-00872-s001.zip › Table S1 and S2.pdf]

Table S1. Primers for cloning into *E. coli* expression vectors

Gene	vector	Primer	Restriction enzymes	Primer sequence (5’-3’)
Rv1506c	pET28a	forward	BamHI	TAGGATCCGTGCGCATTGTCAATGCGGC
		reverse	Hind III	CCTGAAAGCTTTCATGACGCCTTCCTAACCAGA
Rv1505c	pET28a	forward	BamHI	GCGGATCCATGACCAAACCATTGGTAATT
		reverse	Hind III	GTAAGCTTTCATATCTTCCGCAACTCCGTGC
Rv2035	pET28a	forward	BamHI	TAGGATCCATGACACGCCCGCGAACCG
		reverse	Hind III	GCGAAGCTTTCATGGCTGCACCTGGATGC
Rv0976c	pET28a	forward	BamHI	TAGGATCCGTGCGTATCGGAAACTGCTCG
		reverse	Hind III	TAGAAGCTTTCACAACAGGGTCTCCGGGATCT
Rv1185c	pET28a	forward	NdeI	GCGCATATGATGTCCGACTCTTCTGTCTTGTCC
		reverse	HindIII	TATAAGCTTTCAGCCGTCCAACCGGGT
Rv1361c	pET28a	forward	BamHI	TATGGATCCGTGGTGGACTTCGGGGCGTTACC
		reverse	HindIII	ATAAAGCTTTTACCCGGCGGCGGGCACACG
Rv3135	pET28a	forward	BamHI	GTGGGATCCATGGACTACGCGTTCTTACCACCGG
		reverse	HindIII	GCTAAGCTTTCAAGGTGGAGTGCCAGCGGTGTTG
Ag85A	pETSUMO	forward	EcorI	CGAATTCATGGCATTTTCCCGGCCGGGCTTG
		reverse	SacI	ATGAGCTCCTAGGCGCCCTGGGGCGCGGG
Rv1196	pET28a	forward	BamHI	GCTGGATCCATGGTGGATTTCGGGGCGTTACCAC
		reverse	HindIII	TATAAGCTTCTAGCCGGCCGCCGGAGAATGCG

Table S2. Primers for cloning into mammalian expression vectors

Gene	Vector	Primer	Restriction enzymes	Primer sequence (5'-3')
Rv0976c	pVAX1	forward	EcoRI	TAGAATTCGCCACCATGGGCATGCGTATCGGAAACTG
		reverse	PstI	TAACTGCAGCTAGTGATGGTGATGGTGATG CAACAGGGTCTCCG
Ag85A	pVAX1	forward	HindIII	GCGCGCGAAGCTTGCCGCCACCATGCAGCTTGTTGACAGGGTTCGTGGCGCC
		reverse	EcoRI	ATAATGAATTCTCAATGGTGATGGTGATGGGCGCCCTGGGGCGCGGGCCCGGTGTTGGGCG
Rv1185c	pcDNA3.1 (+)	forward	AflII	AAACTTAAGGCCGCCACCATGTCCGACTCTTCTGTCTTG
		reverse	XbaI	CAGTCTAGATCAATGGTGATGGTGATGATGGCCGTCCAACCGGGTG
Rv1196	pcDNA3.1 (+)	forward	AflII	AAACTTAAGGCCGCCACCATGGTGGATTTCGGGGCGTTACC
		reverse	XbaI	CAGTCTAGATCAATGGTGATGGTGATGATGGCCGGCCGCCGGAGAATG
Rv1361c	pcDNA3.1 (+)	forward	AflII	AAACTTAAGGCCGCCACCATGGTGGTGGACTTCGGGGCGTTA
		reverse	XbaI	CAGTCTAGATCAATGGTGATGGTGATGATGCCCGGCGGCGGGCACAC
Rv3135	pcDNA3.1 (+)	forward	AflII	AAACTTAAGGCCGCCACCATGGACTACGCGTTCTTACCACC
		reverse	XbaI	CAGTCTAGATCAATGGTGATGGTGATGATGAGGTGGAGTGCCAGCG
Ag85A	pcDNA3.1 (+)	forward	AflII	AAACTTAAGGCCGCCACCATGCAGCTTGTTGACAGGGTTCG
		reverse	XbaI	CAGTCTAGATCAATGGTGATGGTGATGATGGGCGCCCTGGGGCGCGG
Rv0976c	pcDNA3.1 (+)	forward	AflII	TATACTTAAGGCCGCCACCATGGTGCGTATCGGAAACTGCTCG
		reverse	XbaI	TATCTCTAGATCAATGGTGATGGTGATGATGCAACAGGGTCTCCGGGATCTC
