# Supplementary figures and images for: Protective Efficacy of Subunit Vaccine Expressing Rv0976c Against Tuberculosis
Source: Vaccines (Basel). 2025 Aug 17;13(8):872. doi: 10.3390/vaccines13080872 (PMC12390289; doi:10.3390/vaccines13080872)

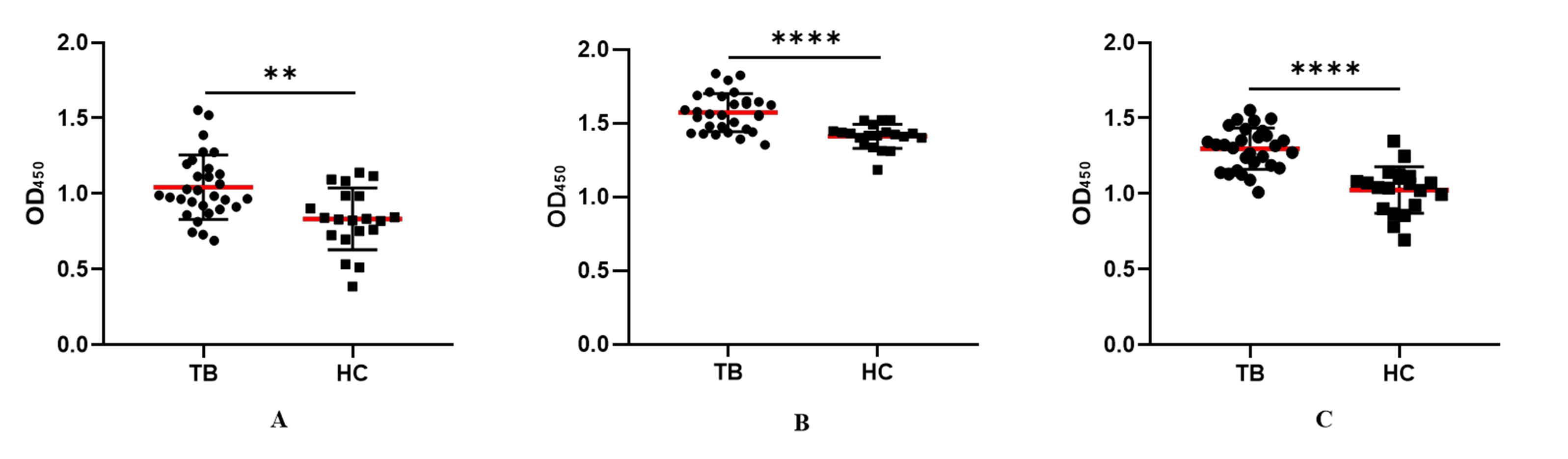

Supplement: Supplementary file 1 [file vaccines-13-00872-s001.zip › Fig S1.tif]

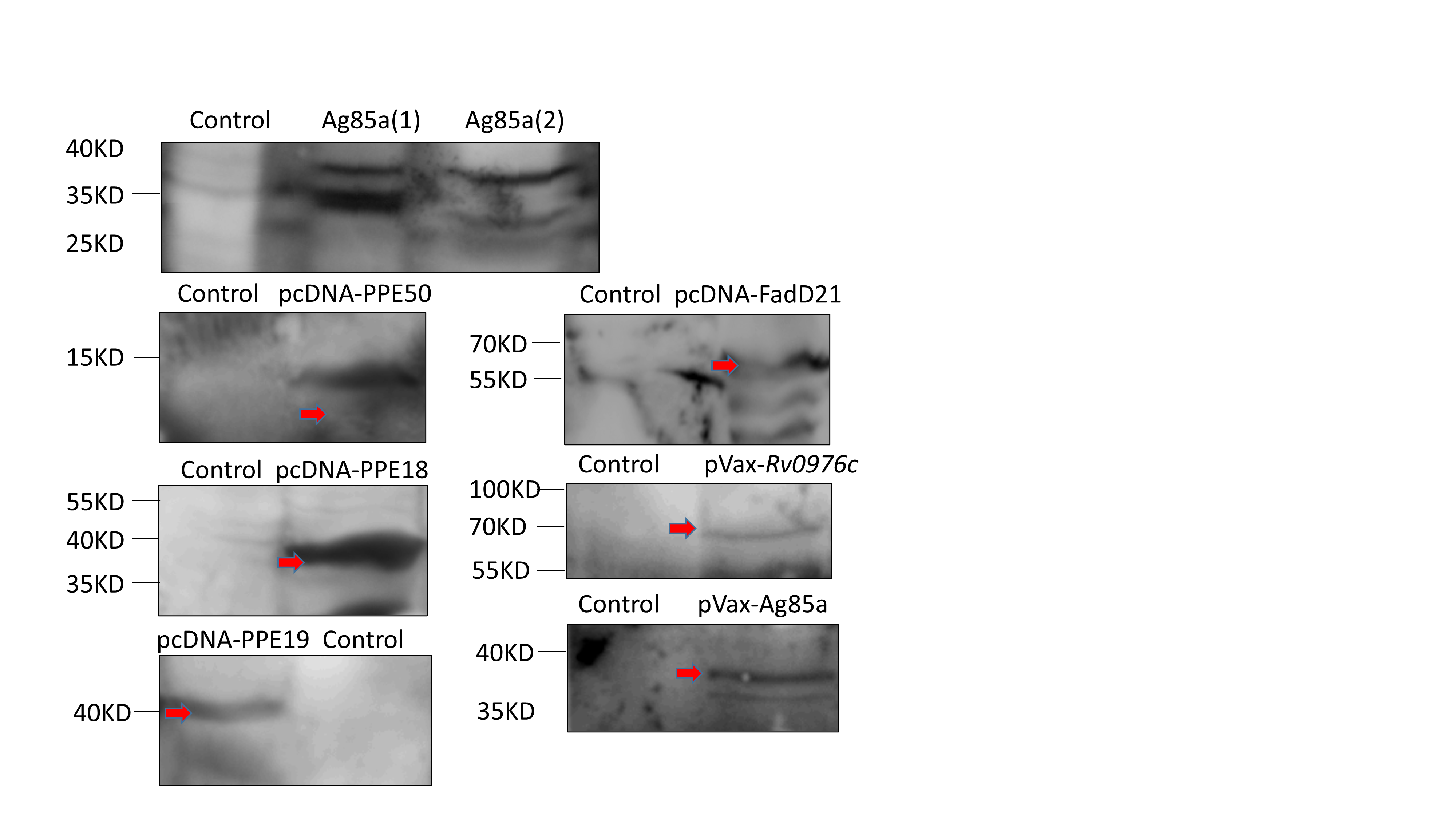

Supplement: Supplementary file 1 [file vaccines-13-00872-s001.zip › Fig S2.tif]
